# Cell Cycle Dynamics of the Nuclear Envelope

**DOI:** 10.1100/tsw.2003.06

**Published:** 2003-03-17

**Authors:** Roland Foisner

**Affiliations:** Department of Medical Biochemistry, Section of Molecular Cell Biology, Vienna Biocenter, University of Vienna, Dr. Bohrgasse 9, A-1030 Vienna, Austria

**Keywords:** BAF, chromatin, chromosomes, higher order chromatin structure, inner nuclear membrane proteins, lamina-associated proteins, lamins, LEM-domain, mitotic phosphorylation, nuclear membrane, nuclear pore complexes, nuclear envelope disassembly, nuclear reassembly, nucleoskeleton, mitotic kinases, Ran GTPase

## Abstract

The nuclear envelope (NE) consists of an inner and an outer membrane, nuclear pore complexes, and the underlying nuclear lamina, a filamentous scaffold structure formed by lamins. The inner membrane is linked to the lamina and chromatin by its integral membrane proteins, such as lamin B receptor (LBR), emerin, and various isoforms of lamina-associated polypeptides (LAP) 1 and 2, which bind lamins and/or chromatin. During mitosis, the NE is disassembled upon phosphorylation of its core components, and the NE is torn apart by a dynein-driven microtubule-dependent mechanism. Nuclear reassembly after sister chromatid separation requires a timely coordinated and dephosphorylation-dependent association of lamin-binding proteins and lamins with chromosomal proteins and targeting of membranes to specific sites on chromosomes. Various chromatin-binding domains in lamina proteins, such as the LEM domain, present in all LAP2 isoforms and in emerin, as well as unique regions in lamina proteins and in specific LAP2 isoforms have been implicated in defined steps of NE reformation. Furthermore, novel mechanisms of membrane fusion involving Ran GTPase are just beginning to emerge.

## COMPONENTS AND POTENTIAL FUNCTIONS OF THE NUCLEAR ENVELOPE

The eukaryotic nucleus is a complex organelle that contains the chromosomes and is the site of DNA replication, RNA transcription and processing, and ribosome assembly. Nuclear functions largely depend on the structural organization of the nucleus and on the formation of a membranous structure, the nuclear envelope (NE), which separates nuclear and cytoplasmic cellular activities. The NE is a double membrane layer composed of two concentric bilayers, the outer nuclear membrane, which is continuous with the endoplasmic reticulum (ER), and the inner nuclear membrane (INM). Outer and inner membranes are separated by a luminal space and are joined at sites, where nuclear pore complexes (NPCs) are inserted into the double membrane system. While the outer membrane is biochemically and functionally similar to the ER and can be considered as a subcompartment of the ER, the inner membrane is clearly distinct in that it contains a specific set of integral membrane proteins[1,2]. The unique protein composition of the INM is thought to be achieved mainly by free diffusion of newly synthesized membrane proteins from the ER through the plane of the outer membrane and the "pore membrane", which connects inner and outer membranes in lateral channels of NPCs, to the inner membrane, where they are retained and stably anchored by specific interactions with intranuclear structures[3,4], such as the lamina and chromatin (reviewed in [5]).

Underneath the inner membrane is a meshwork of nuclear-specific intermediate filaments, termed the nuclear lamina, which provides structural support for chromosomes and maintains nuclear shape and mechanical stability (for review see [6]), spaces NPCs, and is required for key nuclear functions, including DNA replication[7,8.9], and RNA polymerase II-dependent transcription[10]. The core structure of the nuclear lamina is formed by type V intermediate filament proteins, the lamins, which assemble to a meshwork of tetragonally organized 10-nm filaments underneath the INM (so far only shown in *Xenopus* oocyte nuclei)[6,11]. In addition to the lamins, a growing number of lamin-binding proteins, mostly integral membrane proteins of the INM[1] are also considered as genuine components of the nuclear lamina. Interestingly, there is strong evidence, that lamins[12,13] and lamin-binding proteins[14,15] are not restricted to the nuclear periphery but exist throughout the nuclear interior. Their molecular structure and functions are, however, still poorly defined[5,6].

The number and complexity of lamins and lamin-binding proteins has increased during metazoan evolution. While *Caenorhabditis elegans* has only one lamin gene and protein, vertebrates have three lamin genes (*LMNA*, *LMNB1*, *LMNB2*) encoding at least seven distinct isoforms (for review see [16]). Among those, B-type lamins are constitutively expressed in cells throughout development and every cell expresses at least one form of B-type lamins. A-type lamins, comprising lamin A and its smaller splice variant lamin C are only expressed in later stages of development and in differentiated cells. Only B-type lamins contain a stable C-terminal farnesyl modification, which is important but not sufficient for targeting and anchoring the protein to the nuclear membrane[17,18,19,20].

To date, the best characterized vertebrate lamin-binding proteins in the INM include: lamin B receptor (LBR, p58) that has eight transmembrane domains[21], which share extensive homology with sterol reductases[22,23], and interacts with B-type lamins *in vivo* and *in vitro*[24,25,26]. Lamina-associated-polypeptide 1 (LAP-1) comprises three alternatively spliced type II integral nuclear membrane proteins (with a single transmembrane spanning region)[27] that interact with A- and B-type lamins[28,29]. Lamina-associated polypeptide 2 (LAP2) (formerly also called thymopoietin) is a family of six alternatively spliced proteins, of which four (LAP2 $\beta$, $\gamma$, $\delta$, and $\varepsilon$) are type II membrane proteins[30,31]. LAP2$\beta$ has been found to bind lamin B *in vitro*[28] and *in vivo*[32]. LAP2$\zeta$ is a truncated version of the LAP2 membrane proteins that has no transmembrane domain, but neither its cellular localization nor its functions are known. LAP2$\alpha$ is structurally and functionally different from the other isoforms. It shares only the N-terminal 187 amino acids with all the other LAP2 isoforms, but contains a unique C-terminus (506aa) lacking a transmembrane domain[33]. LAP2$\alpha$ is also unique among the LAP2 isoforms as it is located throughout the nucleus[14] and binds specifically A-type lamins in the nuclear interior[15]. Emerin , a type II inner nuclear membrane protein[34,35], and MAN1, a membrane protein containing two transmembrane regions[36], are related to LAP2, as all proteins share a ~40-amino-acid-long highly homologous structural motif, the Lamina-associated-polypeptide Emerin MAN1-domain (LEM domain,) in their N-termini, which comprises a helical turn and 2 large parallel α-helices connected by a 11 to 12 residue loop[37,38]. Emerin binds both A- and B-type lamins *in vitro*[39,40,41,42] and its retention in the NE requires A-type lamins[43,44].

In addition to the lamin-binding proteins other integral proteins of the INM have recently been identified in vertebrates, such as nurim containing 5 predicted transmembrane domains and only few hydrophilic residues[45], Unc-84 and LUMA with three to four predicted transmembrane domains[46,47], a Ring Finger Binding Protein (RFBP), which resembles a type IV phospholipid pump[48], and a new family of spectrin repeat containing type II membrane proteins, Myne-1 and Nesprins[49,50]. Interestingly, Myne-1 has been described to contain an interrupted LEM-like motif and to interact with lamin A[50], but more data are needed to show any functional overlap with LEM proteins.

In addition to the nuclear membrane and the nuclear lamina, NPCs are genuine constituents of the NE. NPCs are large and extremely elaborate structures that mediate bidirectional traffic of macromolecules across the nuclear membranes. They are made of a massive symmetrical framework comprising about 50 different proteins and show an eightfold rotational symmetry[51,52]; 50- to 100-nm-long fibers joined at their distal ends by a ~50-nm ring form a basket like structure extending from the nuclear surface of the core NPCs into the nuclear interior. Nup 153, whose dynamics have been studied fairly well (see below), is a constituent of this nucleoplasmic basket[53]. Two integral membrane proteins have been described in the "nuclear pore membrane": gp210[54,55] and POM 121[56], which may anchor NPCs in the membrane.

The importance of lamina proteins for cell and tissue function has been underlined by several recent findings: functional disruption approaches in *Drosophila*[57], *C. elegans*[58], and cultured mammalian cells[59] revealed that B-type lamins are essential for viability. Targeted disruption of A-type lamins in mice caused muscular dystrophy, loss of adipose tissue, and early death[43]. Furthermore, mutations in the *LMNA* gene or in the gene encoding emerin were linked to heritable human diseases (laminopathies)[60,61,62,63,64]. Emerin mutations cause X-linked Emery Dreifuss Muscular Dystrophy (XL-EDMD)[65], mutations in *LMNA* — an autosomal dominant form of the disease (AD-EDMD)[66], characterized by tendon contractures, wasting and weakness of skeletal muscle, and life threatening cardiac conduction problems. Dominant *LMNA* mutations were also linked to dilated cardiomyopathy with conduction system disease (DCM-CD)[67], limb girdle muscular dystrophy (LGMD1B)[68], and familial partial lipodystrophy (FPLD)[69,70], homozygous defects in *LMNA* to autosomal recessive axonal neuropathy (Chariot-Marie-Tooth disorder type 2, AR-CMT2)[71]. Although these findings point to essential functions of lamina proteins, the molecular mechanisms are still subject to speculation, suggesting roles in structural organization of chromatin in the nucleus, in controlling gene expression directly via transcription factors or indirectly by epigenetic mechanisms, and in cell proliferation.

## NE DYNAMICS DURING INTERPHASE

Like cytoplasmic intermediate filaments, the components of the nuclear lamina, including lamins and lamin-binding proteins, are highly resistant against solubilization in buffers containing nonionic detergent and high salt[28,72], leading to the assumption that the lamina is a highly stable, undynamic structure. Expression of GFP-lamin fusions combined with fluorescence recovery after photobleaching (FRAP) analyses revealed a very slow recovery in late G1 and S-phase cells[13,73,74], supporting the notion of a highly stable structure. FRAP analysis of cells expressing labeled NPC protein POM 121 and lamin B revealed that individual NPCs do not show independent movements relative to each other, most likely due to their tight attachment to the nuclear lamina[74]. These authors observed movement of NPCs and lamina structures in synchronous waves as if they where part of an elastic network, providing compelling evidence for the existence of a flexible but stable lamina – NPC scaffold that supports the nuclear membrane.

Dynamics of GFP-lamin, however, was significantly higher in early G1 cells, when nuclei grow rapidly[13], suggesting rapid incorporation of new subunits into the growing nuclear lamina. Interestingly, expression of a lamin-binding fragment of LAP2β in growing cells was found to inhibit nuclear lamina growth and inhibited progression of cells into S-phase[75]. These results imply two important mechanisms for nuclear lamina assembly in interphase. Firstly, lamina assembly requires interaction of lamin subunits with lamin-binding proteins in the INM[18], a notion also supported by the inability of lamins to form lamina like structures *in vitro*. Secondly, lamina growth is essential for establishing a fully functional nucleus allowing DNA replication. This model is in line with earlier studies showing dependence of DNA replication on an intact lamina in *in vitro* assembled *Xenopus* oocyte nuclei and in cells[7,8,9,76].

Particularly A-type lamins localize transiently in the nuclear interior during G1 phase[15,77]. Thus, A-type lamins are most likely not directly incorporated into the nuclear lamina, but accumulate first in the nuclear interior, where they may be post-translationally processed and/or modified. Similarly, microinjected lamin A or lamin C were found to first accumulate in nucleoplasmic foci to different extent, before the majority was incorporated into the nuclear lamina[78,79]. At least for newly synthesized lamin A, which is post-translationally modified by C-terminal farnesylation and subsequent proteolytic cleavage of its 15 aa C-terminal residues containing the farnesyl group[20], intranuclear localization may be partially linked to processing and maturation of the protein[80,81], but the molecular mechanisms remain unclear.

Despite the very stable nature of interphase lamina and NE structures, there has to be a steady exchange of subunits in the assembled structure, as dominant negative lamin mutants lacking their amino termini rapidly disrupted endogenous lamina structure when expressed in cells[8,76,82]. In agreement with FRAP studies in GFP-lamin B– and GFP-lamin A–expressing cells, showing higher overall stability of B- vs. A-type lamin structures, A-type lamins are more efficiently and more rapidly disassembled by lamin mutants[15,82].

## NE DISASSEMBLY AT THE ONSET OF MITOSIS

The more complex the NE has become during metazoan development the more efficient ways had to be developed to disassemble the NE. In vertebrates, the disassembly of the NE defines the transition between prophase and prometaphase. In lower multicellular eukaryotes *C. elegans* and *D. melanogaster*, the NE is disrupted only partially and/or at later stages of mitosis[16,83], and NE dynamics can be considered as an intermediate between a closed mitosis with no NE breakdown in yeast and a completely open mitosis in vertebrates. NE breakdown involves the depolymerization of the lamina, the fragmentation and removal of the nuclear membranes from condensing chromatin, and the disassembly of NPCs.

Interestingly, very recent data suggest that microtubules themselves help to efficiently disassemble the NE in vertebrates by literally ripping open the NE[84,85] (see Fig. 1). Highly sophisticated live cell imaging analyses of cells expressing fluorescent GFP-fusion proteins[86], immunofluorescence[87], and electron microscopy[88] revealed that duplicated centrosomes attach to the NE in nuclear invaginations. While previous studies have suggested that the NE ruptures at sites of invaginations due to pushing of microtubules[87], recent studies clearly showed that the NE seemed to be pulled apart at sites distal to the invaginations[86,88]. By

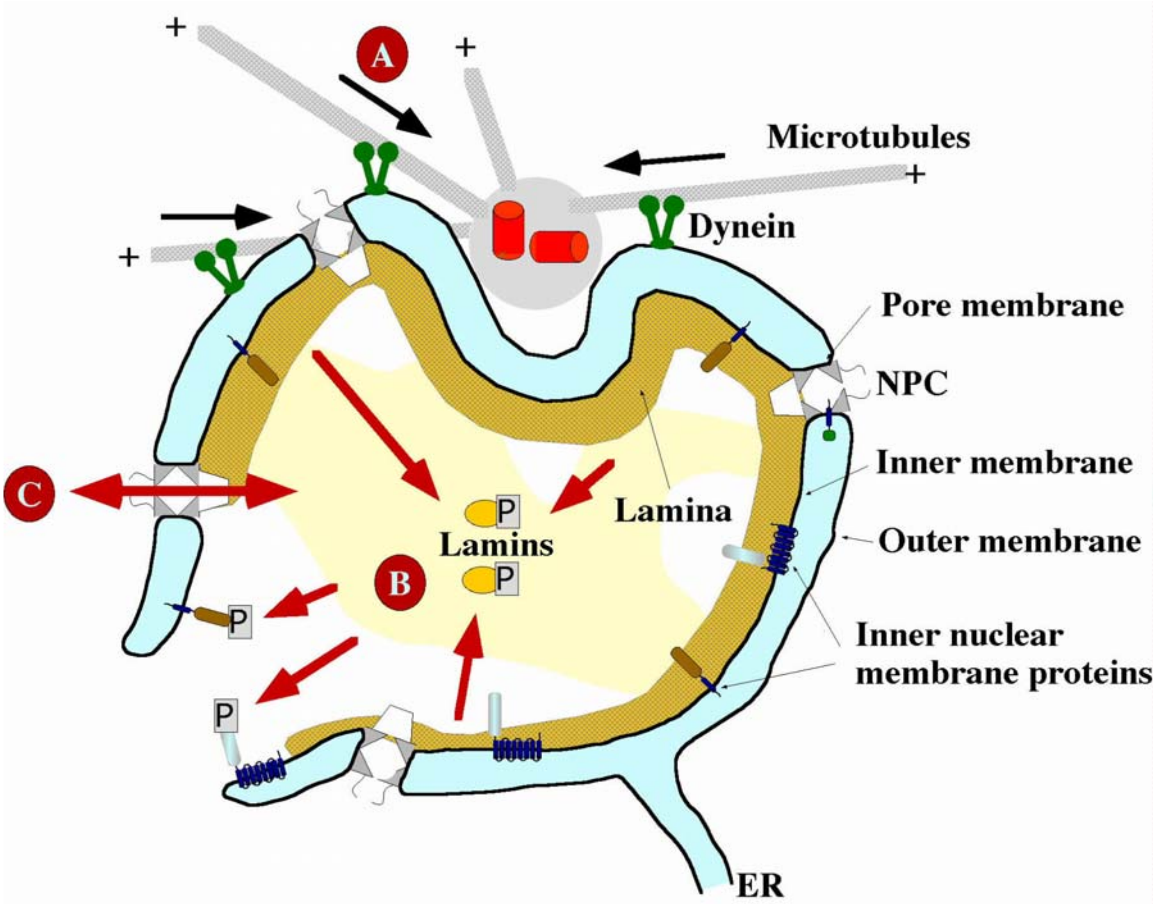

**FIGURE 1**. Molecular mechanisms of NE disassembly at the onset of mitosis. Arrows denote specific mechanisms known to be involved in NE disassembly. (A): Dynein/dynactin complexes attach at the NE and migrate towards the minus ends of microtubules, assembled from cytoplasmic centrosomes in NE invaginations, and thus, generate a tension that tears the NE open at sites distal to the centrosomes. (B): Lamins and Lamin-binding proteins are phosphorylated by mitotic kinases and dissociate from the lamina and chromatin. (C): NPCs are partially disassembled and become leaky, allowing larger molecules to passively diffuse in or out of the nucleus.

pulling NE components towards the centrosomes, membranes seem to be torn open due to tension. The mechanical force is provided by a minus-end-directed microtubule motor dynein, which is recruited to the NE at late G2 phase[88]. Overexpression of a dynactin component, which usually mediates interaction of dynein with membranes, was found to delay NE breakdown, probably by interfering with the attachment of endogenous dynein to the membranes. The interaction partner for dynein at the NE remains, however, obscure. It also has to be stated, that the microtubule-dependent NE rupture only facilitates NE breakdown, but is not essential for this process, as nuclear disassembly can also occur in the absence of microtubules.

In view of this novel model for NE disassembly, the contributions of other NE components or other mechanisms to NE breakdown (Fig. 1) appear less clear. The disassembly of NPCs provides one example for another potentially important process in NE breakdown. Using fluorescent dextran dyes, large enough to be excluded from the nucleus, Terasaki et al.[89] could distinguish two phases of nuclear accumulation of dextran during NE breakdown in starfish oocytes, an initial slow uniform increase followed by a massive wave. The slow nuclear translocation of dextran has been attributed to partial NPC disassembly causing leakiness, while the massive wave was correlated with the formation of holes in the NE. This observation suggested that loss of NPC function may precede NE rupturing and may thus allow diffusion of larger protein complexes into the nucleus, which may initiate disassembly. In line with this hypothesis, distinct stages of NPC disassembly have been visualized by field emission scanning electron microscopy during mitosis in early *Drosophila* embryos, including the release of the central transporter and the removal of the cytoplasmic ring subunits followed by removal of star rings[90]. On the other hand, studies in sea urchin embryonic nuclei have shown that blocking NPC function by wheat germ agglutinin prevented NE breakdown, suggesting that active nuclear transport is required for initiation of NE breakdown[91]. A second possibility, however, is that binding of wheat germ agglutinin to nucleoporins directly blocks NPC disassembly.

In mammalian cells, transport of mitotic cyclin-dependent kinases through NPCs[92] may be required for efficient phosphorylation of lamins and lamin-binding proteins, which then trigger the disassembly of lamins and the dissociation of proteins from chromatin (for review see: [6,33,93]). Phosphorylation might be a prerequisite for microtubule-dependent membrane rupture, as phosphorylation-dependent disassembly of lamin structures or dissociation from chromatin may destabilize the NE (Fig. 1). In line with this notion, cells expressing lamin mutants that can not be phosphorylated by mitotic kinases did not efficiently disassemble the NE[94]. Furthermore, lamin A was found to dissociate from the nuclear lamina into the nucleoplasm in early prophase, before it was massively released into the cytoplasm upon NE rupture[87]. This suggests that (phosphorylation-dependent) disassembly of lamin A structures occurs before the NE becomes leaky regardless of the mechanism of NE rupturing.

Phosphorylation of LBR[95,96] and LAP2[14,28] likely triggers the dissociation of membranes from chromatin. B-type lamin subunits often remain associated with membranes due to their C-terminal farnesyl modification, and probably also due to an interaction with LBR throughout mitosis[24]. Immunofluorescence and live cell imaging studies suggested that the nuclear membranes merge into the ER and nuclear membrane proteins diffuse freely throughout the mitotic membrane structures[97,98]. Other studies involving cell lysis and membrane fractionation suggested domain specific vesicularization of the nuclear membrane into different populations of vesicles containing different sets of proteins from the inner nuclear membrane, the pore membrane or the outer nuclear membrane/ER[99,100]. However, in these studies membrane vesicularization and fragmentation might have been caused by cell homogenization (discussed in [101]). Eggs of *Xenopus* or sea urchin, on the other hand, which contain huge stockpiles of membranes required to support rapid cell divisions, may contain different types of membranes that support nuclear assembly[102,103,104].

## POSTMITOTIC REASSEMBLY OF THE NE

Open mitoses poses problems, because NE and nuclear structure reassembly has to proceed in a tightly regulated manner after chromatid separation, ensuring that the interphase organization of chromatin can be reestablished in daughter nuclei[16].

Numerous studies have shown that the assembly of the NE and the establishment of nuclear and chromatin organization after cell division involves the targeting and assembly of lamins and lamin-binding proteins in a temporally and spatially regulated manner.

### Interactions of NE Components with Chromosomes

In principle, the targeting of NE components to the chromosomal surface could be mediated by any protein that is able to interact with DNA or chromosomal proteins. Lamins can interact with and assemble around chromatin *in vitro.* This assembly involves their rod domain[105,106] and/or their C-terminal tail domain that binds to core histones[107,108]. In addition, many lamin-binding proteins were shown to interact with DNA and/or chromosomal protein. LBR interacted directly with DNA[26,109] and bound to human heterochromatin HP1-type chromodomain proteins[110,111] and to histones H3/H4 in a histone acetylation-dependend manner[112,113]. In cross-linking studies LBR was also found to associate with chromatin-associated HA95, a nuclear protein with high homology to the nuclear A-kinase anchoring protein AKAP95[114]. LAP2 proteins contain several chromatin and/or DNA binding domains, which are either common to all or unique to some isoforms. The LEM domain (amino acids 111–152) in the constant N-terminal region common to all LAP2 proteins (see also Fig. 3), was found by yeast two hybrid assays and by biochemical studies to interact with the chromosomal protein **B**arrier-to-**A**utointegration **F**actor (BAF)[115,116]. BAF is an 89-residue, highly conserved protein in multicellular eukaryotes[117] that binds double-stranded DNA without sequence specificity forming nucleoprotein complexes (dodecamers) between DNA molecules[118]. The LEM domain in emerin also bound BAF[39], that in MAN1 has not been experimentally tested yet. Moreover, the N-terminal 50 residues of the LAP2 constant region were found by structural studies to contain a LEM-like motif that bound DNA[37] and an N-terminal 85 residues LAP2 fragment containing the LEM-like motif associated with chromosomes *in vitro*[32]. In addition to the common N-terminal chromatin binding domains, a DNA binding region has been identified in the LAP2ß-specific region[119], and a chromosome association region in the unique C-terminus of the nucleoskeleton-associated isoform LAP2$\alpha$[120,121]. Similar to LBR, LAP2ß has also been identified by cross-linking experiments to associate with HA95[114]. Thus a complex sequence of interactions between NE components and chromatin is likely involved in the reformation of fully functional daughter nuclei after sister chromatid separation.

## Potential Mechanisms of NE Assembly

In the past years, several labs have investigated the detailed timing of the accumulation of different NE proteins relative to each other at decondensing chromosomes by immunofluorescence and time-lapse microscopic studies in fixed and living cells. By transient expression of GFP-tagged NE proteins Haraguchi et al.[122] could observe membrane targeting to chromosomes 5 min after metaphase-anaphase transition, involving accumulation of LBR and emerin. This was followed by NPC assembly between 5 to 7 min after metaphase-anaphase transition, involving early accumulation of Nup153 and p62 NPC protein. Nuclear import activity could be observed as early as 8 min after metaphase-anaphase transition, suggesting that nuclear-transport-dependent steps may be important for later stages of NE assembly and/or for NE growth. LAP2$\beta$ was also found to accumulate at chromosomes early[13,28,123] at around the same time as emerin[124]. Finally, we have shown that LAP2$\alpha$ assembled around chromosomes even earlier than LAP2$\beta$[121], suggesting that it is the first protein among the NE/nucleoskeleton components to associate with chromosomes during assembly. Concerning the lamins, recent studies applying GFP tagged lamins have shown that assembly of B- and A-type lamins followed clearly different pathways. B-type lamins associated with chromosomes at early stages of NE reformation, yet LAP2$\beta$ associated earlier, as it had nearly completely accumulated around chromosomes, when lamin B was detectable on the chromosomal surface[13]. Nevertheless, these

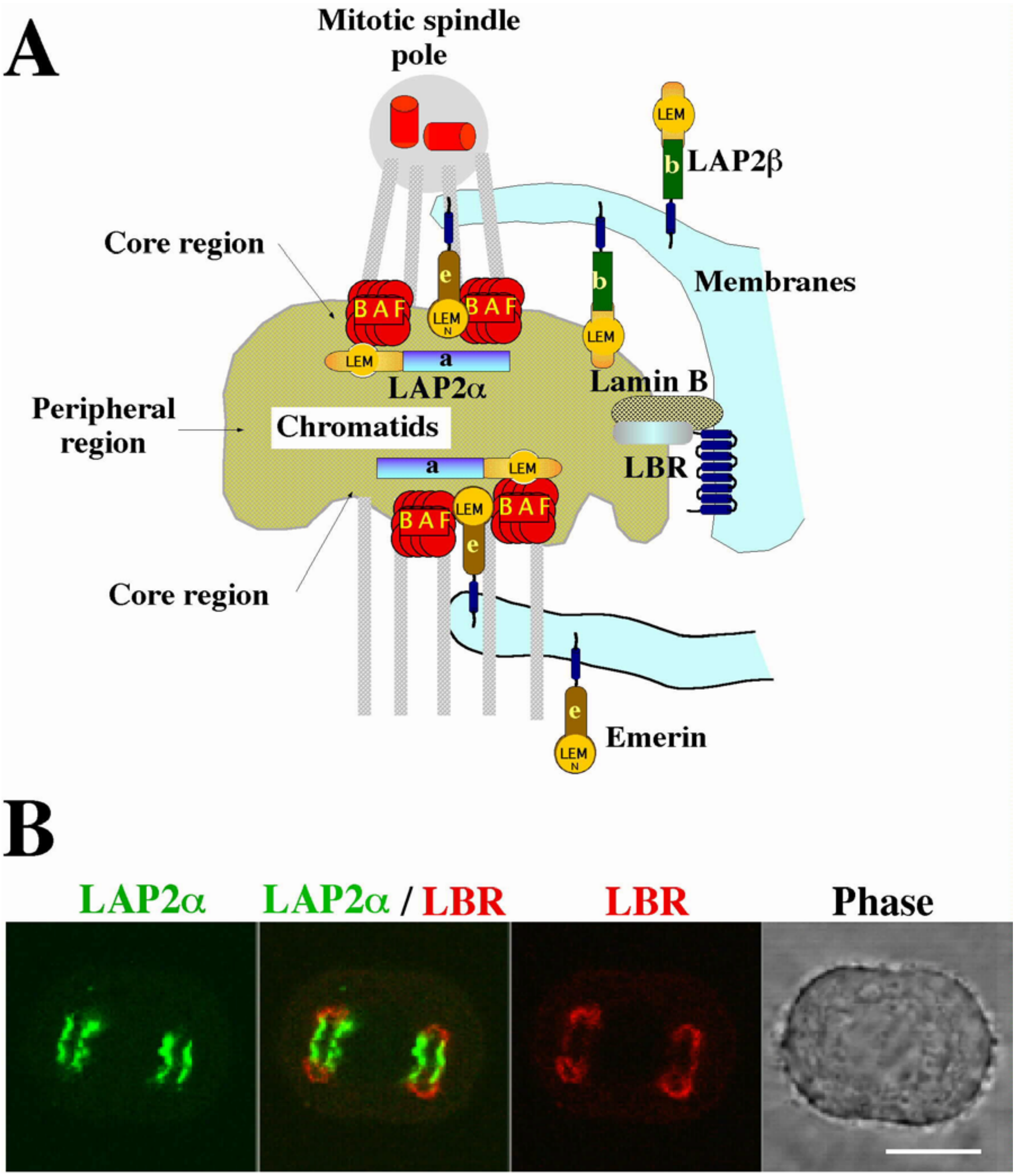

**FIGURE 2.** Differential attachment sites of proteins at chromosomes during nuclear assembly. (A): Schematic representation of a half-spindle and separated sister chromatids at anaphase, showing preferred attachment of LAP2α, BAF, and emerin at core regions, and LBR, lamin B and probably LAP2β at peripheral regions of decondensing chromosomes. LEM domains and transmembrane regions of proteins are indicated; LAP2α is labeled with a, LAP2β with b, and emerin with e. (B): Confocal immunofluorescence image of a cell in anaphase expressing YFP-labeled LAP2α (green) and CFP-labeled LBR (red), and phase contrast image of the same cell. Merge of red and green stain is shown to visualize the different localization of proteins at chromosomes during nuclear assembly in anaphase. Image was kindly provided by Thomas Dechat. Bar represents 10 μm.

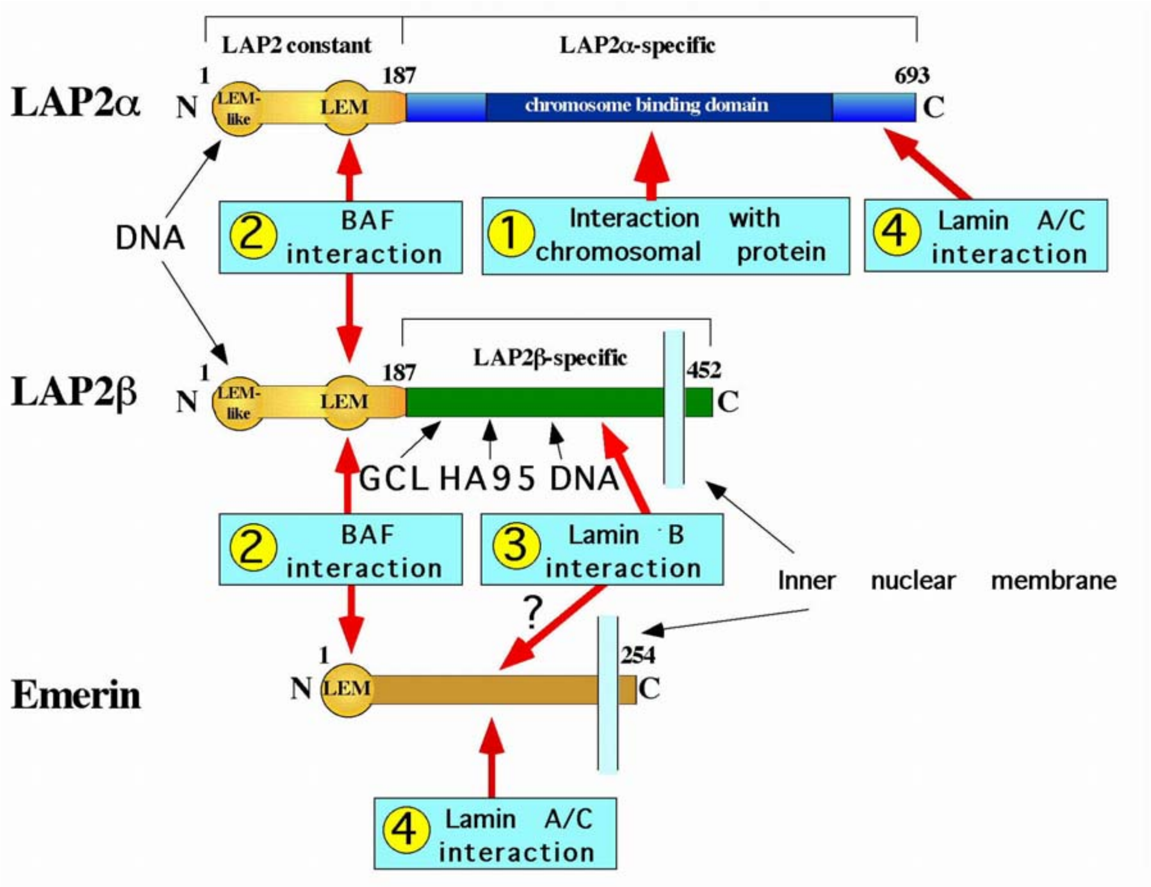

**FIGURE 3.** Specific interaction of LEM-domain proteins during nuclear assembly. Domains in LAP2 proteins and in emerin are shown. Numbers in boxes indicate the sequence of interactions occurring during NE assembly after mitosis, and red arrows denote interaction domains in the proteins. Black arrows denote interactions, which have not been shown to occur at specific time points of assembly and/or may be important for stabilizing interactions of assembled NE with chromatin in interphase (HA95, DNA) or controlling gene expression (GCL[169]) For details, see text.

studies indicated that a significant fraction of lamin B may access chromosomes directly prior to NE assembly without being imported through NPC. The bulk of lamin A, on the other hand, seemed to translocate through newly assembled NPCs and to accumulate in the nuclear interior rather than the nuclear periphery[13], where they may interact with intranuclear LAP2α structures in late telophase/G1 phase[14,15]. Thus, the order of assembly of these proteins may be: LAP2α, followed by membrane proteins LAP2β, LBR, and emerin, and the NPC proteins NUP 153 and POM 121, followed by lamin B, followed by lamin A.

Another intriguing difference in the dynamic behavior of NE proteins during nuclear assembly was observed in regard to the sites, where proteins associate with chromosomes (Fig. 2). LAP2α accumulated at “core regions” of the bulk of decondensing chromosomes, which are the regions next to the spindle pole- and midspindle areas[5,14] (Fig. 2B), emerin was first seen at more peripheral sites but accumulated then also at the core regions[122,125], when also a subfraction of lamin C was detectable there (our unpublished observation). LAP2β, lamin B, and particularly LBR accumulated initially at the peripheral chromosomal regions before they formed a continuous rim around the decondensed chromatin[13,97,122,124,125]. This strongly suggested that the chromosome association of these different sets of proteins involves different mechanisms.

The different behavior of LAP2α and LBR can be easily explained by their interactions with different proteins on chromosomes, such as HP1 and histones for LBR vs. BAF for LAP2α (see above). The different dynamics of LAP2α, LAP2β, and emerin in regard to timing and initial site of chromosome association seem, however, less obvious, as all proteins share the LEM domain which mediates interaction with chromosomal BAF.

Several recent observations might help to explain this phenomenon. Firstly, we could show, that the association of LAP2α with chromosomes at early stages of assembly, required the C-terminal chromatin binding domain that is unique for LAP2α, while the N-terminal LEM-domain was dispensable for this interaction[121] (Fig. 3). Secondly, fragments that contained the LEM domain but lacked the α-specific chromatin-binding region did not associate with chromosomes *in vivo* and *in vitro*[120,121]. Thirdly, *in vitro* nuclear assembly assays showed that initial binding of full length LAP2α is required for membrane assembly around chromosomes, while chromosome binding fragments of LAP2α lacking the LEM domain inhibited assembly[120]. Thus one can envisage the following model for the initial stages of assembly (Fig. 3): LAP2α associates first with chromosomes via its C-terminus and this interaction does not depend on or involve the N-terminal LEM domain. This association may trigger conformational changes on the chromosomal surface and/or target cytoplasmic BAF to the chromosomes and/or induce post-translational modification of BAF or other chromosomal proteins, thus allowing interaction of the LEM domain with BAF. We propose that LAP2β and emerin are then recruited to “active” BAF by their LEM domains and form membrane structures around the chromosomal surface. This model is supported by three recent findings. Firstly, BAF localized to the same core regions on chromosomes, where LAP2α accumulated[125]. Secondly, expression of a mutant BAF that did not bind to the LEM domain and to DNA and failed to localize at core domains inhibited assembly of emerin, LAP2β, and lamin A, but not lamin B, at the NE[125]. Unfortunately, LAP2α has not been tested yet in these cells. Thirdly, addition of BAF to *in vitro Xenopus* nuclear assembly reactions deregulated chromatin condensation, causing enhanced chromatin decondensation at low concentrations and a block of decondensation and nuclear assembly at higher concentrations[126].

## Contribution of Individual Proteins to NE Assembly

NE assembly includes a complex, highly regulated, and timely coordinated sequence of interactions. To analyze the specific contributions and importance of different NE components and their interactions for nuclear assembly, several labs have expressed mutants in cells or have added mutant proteins to *Xenopus* and mammalian *in vitro* nuclear assembly assays, or have immunodepleted specific NE proteins from *in vitro* assembly extracts. As mentioned above, addition of wild type BAF to *Xenopus* egg extracts[126], or expression of BAF mutants lacking emerin- and DNA binding in cells[125] interfered with NE assembly. Similarly, addition of LAP2α mutants missing the LEM domain to mammalian nuclear assembly reactions[120], or addition of N-terminal LAP2 constant region to *Xenopus* egg extracts[127] inhibited assembly of nuclear membranes. However, expression of the LAP2 constant region in cells had no effect[120,121]. Immunodepletion of LBR from membrane vesicles of avian cells inhibited binding to chromosomes[128] and addition of LBR antibodies to sea urchin egg extracts affected nuclear assembly[129].

Interestingly, studies in the *Xenopus* nuclear assembly system have revealed two distinct vesicle populations[104], which contain different sets of membrane proteins, and recruitment of these vesicles in an ordered fashion was essential for nuclear membrane assembly[103]. This, however, may be a specific feature in oocytes that contain huge stockpiles of membranes. Taking together, interfering with functions of most NE/chromatin proteins significantly affects NE assembly, suggesting that all these proteins are essential for nuclear assembly during a specific stage or in a specific cell system.

The contribution of lamins to NE assembly has been controversial[130]. While immunodepletion of lamins from *Xenopus in vitro* nuclear assembly extracts did not inhibit NE formation[9,131], other studies using *Drosophila*, mammalian, and *Xenopus* extracts showed that immunoadsorption of lamins inhibited NE assembly[132,133,134]. The different results were most likely caused by the different efficiencies in depleting and/or deactivating lamins by antibodies or might be attributed to the continued presence of aggregated lamins in the assembly reaction. Lopez-Soler et al. have recently added a peptide representing the C-terminal domain of *Xenopus* lamin B3, which inhibited lamin interactions and lamina assembly, to nuclear assembly reactions and found inhibition of membrane assembly around chromatin[135]. Conversely, inhibition of lamin B assembly in HeLa cells by inhibiting its protein phosphatase 1 (PP1)-dependent dephosphorylation did not block the assembly of the nuclear membrane and of lamin A, but induced apoptosis in G1 phase[136,137]. Thus, although a polymerized lamina (containing lamin B) is not important for assembly of the nuclear membrane, it is essential for cell viability in interphase, as also shown by functional disruption of B-type lamins in *Drosophila*[57] and *C. elegans*[58]. Taking all these experiments together, one may conclude that lamins may be important for nuclear membrane assembly, a typical lamina however, is not.

### Assembly of NPCs

As for NPC assembly, basket-associated Nup 153 and the pore membrane protein POM 121 are the first NPC proteins to associate with chromatin, whereas pore membrane protein gp210 and Tpr, which associates with the nuclear surface of NPCs, accumulate later[123]. The sequential recruitment of NPC proteins may reflect the appearance of discrete intermediates in the assembly of mature NPCs *in vitro*[138]. Thus, binding of Nup 153 or POM 121 to chromatin could define sites of NPC assembly, which subsequently may recruit additional hierarchies of NPC proteins. In line with such a hypothesis, NPCs assembled in the absence of Nup 153 from immunodepleted extracts lacked several basket structures, were not stably anchored in the NE, and were defective in the importin-mediated transport[139]. In other studies Nup 153 was found to interact with lamin LIII, and Nup153 assembly at chromosomes was dependent on the formation of a lamina[140]. Thus, Nup153 may be important for linking the NPC to the lamina. Strikingly, however, Nup153 fluorescence recovered much faster than those of other NPC proteins[74], indicating that Nup153 undergoes a rapid exchange between intranuclear and NPC associated pools. Thus Nup 153 may have different roles during assembly and in mature NPC, where it may mainly be involved in transport. The presence of zinc-finger motifs in Nup153 that may mediate DNA interaction[141] may facilitate its early association with chromosomes during nuclear reassembly in a membrane-independent manner.

## REGULATION OF NE DYNAMICS

### Phosphorylation-Dependent Mechanisms

The rapid disassembly of the NE at the onset of mitosis is driven by mitotic phosphorylation of lamins[142], LAP1 and LAP2[14,28,33], probably LBR[95,96,109,143,144], NPC proteins[145,146], and most likely other NE components. For lamins, $p34^{cdc2}$ kinase, has been identified as the most prominent mitotic kinase involved in the disassembly of the lamina *in vivo* and *in vitro*[94,147,148,149,150], and ectopically expressed lamin A with mutations in the *cdc2* phosphorylation sites failed to be disassembled during mitosis. There are also other kinases known to be involved[151], such as PKC[152,153]. Membrane proteins are most likely also targets for mitotic p34$^{cdc2}$ kinase, but this has not been confirmed by mutating the respective phosphorylation sites in the proteins and analyzing the effect on assembly/disassembly *in vivo*. LBR was also shown to be phosphorylated by a serine/arginine kinase[95,144] that modulated interaction of LBR with other nuclear proteins.

Nuclear reassembly requires phosphatase activity and, at least for B-type lamins, has been shown to involve phosphatase PP1[154]. PP1 is targeted to the NE by a membrane protein of the ER and the NE, protein A-kinase anchoring protein (AKAP149)[155]. PP1 recruitment from chromosomes to membranes at the nuclear periphery in telophase is a prerequisite for assembly of B-type lamins. Inhibition of PP1 association with AKAP149 by a peptide containing the PP1 binding domain of AKAP149 resulted in lack of assembly of B-type lamins and apoptosis[136,137]. These findings suggest that lamin B assembly is dependent on the presence of membranes at the chromatin surface and confirms time-lapse microscopic studies showing that lamin B assembly starts after LAP2β-containing membranes have bound to chromosomes[13]. Interestingly, assembly of A-type lamins was not effected by the peptide, supporting other studies which show different pathways of assembly of A- and B-type lamins after mitosis[13,15].

## Mechanisms Involved in Later Stages of NE Assembly

While early stages of NE assembly, such as membrane targeting to chromosomes, are energy independent and may mostly depend on protein dephosphorylation, the events causing membrane fusion are less well known. GTP hydrolysis was known to be required for vesicle fusion for several years[156,157], but only more recent studies in *Xenopus* egg extracts have shed light on the molecules involved. Both Ran GTPase activity and RCC1, the nucleotide exchange factor for Ran, have been found to be essential for extensive membrane fusion on the chromatin surface[158]. As beads coated with Ran-GDP were also surrounded by a membrane containing functional NPC in a RCC1- or GTPase activity-dependent manner, this process seems to be independent of chromatin[159]. It is not known, however, how membranes were targeted to the bead's surface, whether a stable nuclear lamina assembled, and whether INM proteins were accumulated in these studies. The involvement of Ran GTPase in NE assembly has also been confirmed *in vivo* by RNAi mediated downregulation of Ran or RCC1 in *C. elegans* embryos[160].

Intriguingly, importin-β, a Ran effector in nucleocytoplasmic transport[161,162], has been suggested to mediate Ran-dependent NE assembly in an importin-α-independent manner[163]. In this model, importin-β interacting with FxFG repeat containing nucleoporins may be targeted to chromosomes due to its interaction with Ran GTP, which is generated by chromosome bound GTP/GDP exchange factor RCC1. Since binding of importin-β to Ran-GTP decreases its affinity for nucleoporins, they may be released locally at the surface of chromosomes and facilitate NPC and NE assembly. Since Ran-regulated interactions of importin-ß control also nuclear transport[161] and mitotic spindle assembly[164,165,166], changes in localization, concentration, and interactions of RanGTP/RanGDP and importin-β may help coordinate spindle assembly/disassembly with reassembly of the NE and with initiation of nucleocytoplasmic transport. The potential function of importin-β in NE assembly was also confirmed in *Drosophila* expressing an importin-β mutant that lacked RanGTP binding activity[167].

The molecular components involved in membrane fusion are just beginning to be discovered. These may include both components previously known to be involved in fusion of Golgi and ER membranes, such as AAA-ATPase p97-p47 adaptor complexes during NE growth, and complexes not previously implicated in membrane fusions, such as p97/Ufd1-Npl4 adaptor complexes. Interestingly, the latter complexes have been implicated in early NE fusion events[168]. Assuming that ER and INM are interdispersed in mitotic stage, different adaptors responsible for fusion of ER and NE membranes would ensure that both membrane systems, ER and NM, are processed in different ways upon exit from mitosis. It is intriguing to speculate that INM proteins could be involved in such a mechanism by recruiting specific adaptor-p97 complexes.

## ACKNOWLEDGEMENTS

I want to thank Thomas Dechat, Vienna Biocenter, for providing immunofluorescence images in Fig. 2. I am also grateful to Sylvia Vlcek, Vienna Biocenter, for her valuable comments on the manuscript. Work in the author's laboratory was supported by grants from the Austrian Science Research Fund (FWF) No. P15312, from the Jubiläumsfonds of the Austrian National Bank, and from the "Österreichische Muskelforschung" to RF.
